# ASSOCIATION OF BIOCHEMICAL, CLINICAL, AND ANTHROPOMETRIC PARAMETERS WITH FUNCTIONAL PERFORMANCE OF OLDER WOMEN

**DOI:** 10.1093/geroni/igad104.2453

**Published:** 2023-12-21

**Authors:** Silvana Funghetto, Erika Ferreira, Mateus Leite, Alessandro Oliveira de Silva, Marina Morato Stival Lima, Luciano Ramos de Lima, Yuri Barbalho Sousa Barbalho, Cris Renata Volpe

**Affiliations:** University of Brasilia- Campus Ceilândia- UNB-Brasil, Brasília, Distrito Federal, Brazil; University of Brasilia, Brasília, Distrito Federal, Brazil; University of Brasilia, Brasília, Distrito Federal, Brazil; Center University of Brasilia, Brasília, Distrito Federal, Brazil; University of Brasilia, Brasilia, Distrito Federal, Brazil; University of Brasilia, Brasilia, Distrito Federal, Brazil; University of Brasilia, Brasilia, Distrito Federal, Brazil; University of Brasilia, Brasilia, Distrito Federal, Brazil

## Abstract

Increased longevity is a widespread worldwide trend, evidenced by demographic and epidemiological data, especially with regard to females, who live an average of seven years longer than males. The aging process is associated with a progressive decline in muscle mass, strength, power, impaired balance, altered heart function and vascular function. These changes negatively affect exercise capacity, increase the risk of cardiovascular disease, and have implications for physical function and the risk of falling. This is particularly relevant among women diagnosed with impaired functional performance. The aim of this study was to determine an association of biochemical, anthropometric, metabolic and inflammatory parameters according to the functional performance of older women aged 62 to 75 years attended for in primary health care. Statistical results showed that the 116 older women (56.86%) identified with impaired functional performance had a pro-inflammatory marker, high anthropometric indices and body composition, impaired cognitive performance and lower muscle strength as older women ( 88 ) with normal functional performance (43.14%). Age, waist-to-height ratio and elevated IL-6 and muscle strength significantly decreased the risk of impaired functional performance. These important findings demonstrate the need for further research documenting the underlying processes and risk factors for preserving functional capacity, carrying out life activities and decreasing the emergence of illnesses. The potential benefits of this approach provide a basis for developing individualized health promotion interventions in the management of this population of older women.

